# Antifibrotic Agents in Rheumatoid Arthritis-Associated Interstitial Lung Disease: A Systematic Review and Meta-Analysis

**DOI:** 10.3390/life13122318

**Published:** 2023-12-10

**Authors:** Ji Hoon Jang, Junghae Ko, So Young Jung, Dae-Wook Kim, Ju Hyun Oh, Tae-Jung Kim, Joo Hun Park, Miyoung Choi, Jae Ha Lee

**Affiliations:** 1 Division of Pulmonology and Critical Care Medicine, Department of Internal Medicine, Haeundae Paik Hospital, Inje University College of Medicine, Busan 48108, Republic of Korea; saturn80396@gmail.com; 2 Division of Endocrinology, Department of Internal Medicine, Haeundae Paik Hospital, Inje University College of Medicine, Busan 48108, Republic of Korea; arrioph1@gmail.com; 3 Department of Dermatology, Haeundae Paik Hospital, Inje University College of Medicine, Busan 48108, Republic of Korea; docjsy@hanmail.net; 4 Department of Orthopedic Surgery, Haeundae Paik Hospital, Inje University College of Medicine, Busan 48108, Republic of Korea; wook0912@naver.com; 5 Department of Pulmonary and Critical Care Medicine, Sanggye Paik Hospital, Inje University College of Medicine, Seoul 01757, Republic of Korea; angle5018@naver.com; 6 Department of Hospital Pathology, Yeouido St. Mary’s Hospital, The Catholic University of Korea, Seoul 07345, Republic of Korea; kimecho@catholic.ac.kr; 7 Department of Pulmonary and Critical Care Medicine, Ajou University School of Medicine, Suwon 16499, Republic of Korea; jhpamc@naver.com; 8 Division of Health Technology Assessment Research, National Evidence-Based Healthcare Collaborating Agency (NECA), Seoul 04933, Republic of Korea; myhams95@gmail.com

**Keywords:** antifibrotic agents, rheumatoid arthritis, interstitial lung disease, meta-analysis

## Abstract

Background: Rheumatoid arthritis-associated interstitial lung disease (RA-ILD) is a common extra-articular clinical manifestation of rheumatoid arthritis (RA) that has negative impacts on morbidity and mortality. In addition, there has been no proven treatment for RA-ILD to date. Thus, we planned a meta-analysis of a literature search to confirm the clinical effects of antifibrotic agents in RA-ILD patients. Materials and Methods: We conducted the literature search in Ovid MEDLIVE^®^ databases, Cochrane Library databases, EMBASE, and KoreaMed and identified references elucidating the role of nintedanib or pirfenidone in adult patients with RA-ILD. Among the identified studies, those with comparative interventions, complete results of clinical trials, and available full text were included in the analysis. The primary outcome was the effect of the antifibrotic agent on disease progression in RA-ILD patients assessed with a mean difference in the change of forced vital capacity (FVC) and the proportion of patients with an increase in percent predicted FVC of 10% or more over 52 weeks. Analysis for heterogeneity was assessed through I^2^ statistics. Meta-analysis with a fixed effect model was performed on changes in FVC. Results: A total of 153 articles were identified through database searches, of which 28 were excluded because of duplication. After additional screening, 109 studies were selected with full text and two articles qualified for analysis according to the set inclusion and exclusion criteria. As a result, two randomized controlled studies were selected, comparing nintedanib and pirfenidone to placebo, respectively. The meta-analysis revealed that antifibrotic agents showed a significant reduction in FVC decline compared to placebo in patients with RA-ILD (mean difference, 88.30; 95% CI, 37.10–139.50). Additionally, there were significantly fewer patients experienced an increase in percent predicted FVC of 10% or more in the antifibrotic agent group compared to the placebo group (Odds ratio 0.42; 95% CI 0.19–0.95, *p* = 0.04). There was no significant heterogeneity between the two included studies (χ^2^ = 0.35, *p* = 0.0007, I^2^ = 0%). Conclusions: The meta-analysis suggests that nintedanib and pirfenidone may have clinical utility in reducing disease progression in patients with RA-ILD. Further research is needed to confirm the clinical benefits of antifibrotic agents in RA-ILD.

## 1. Introduction

Rheumatoid arthritis (RA) is one of the most common systemic autoimmune disease, characterized by a chronic inflammatory arthritis and eventual joint destruction affecting 0.5–1% of the population worldwide [1]. RA can involve not only joints but also various tissues and organs throughout the body. Extra-articular involvements of RA occur in approximately 40% of patients with RA and can occur both early in the disease and during the course of the disease [2].

The most common extra-articular involvement is pulmonary involvement, with varying prevalence ranging from 15% to 58%, depending on the study among RA patients during the course of the disease [3,4,5]. Among the various forms of pulmonary involvement, interstitial lung disease (ILD) is the most common, and it can manifest either as a subclinical interstitial lung abnormality (ILA) or as a clinically significant symptomatic ILD [6]. The overall prevalence of RA-associated ILD (RA-ILD) varies from 10% to 50% across the literature, depending on the definition of ILD and screening tools [6,7]. The prevalence of clinically significant symptomatic RA-ILD is 7–10%, accounting for 10–20% of all mortality associated with RA [6,8,9,10]. Previous studies have reported that patients with RA-ILD have a 2 to 10 times higher mortality rate than those without RA-ILD [6,8,9].

In RA-ILD, the usual interstitial pneumonia (UIP) pattern is the most common histologic and radiologic feature with a prevalence that ranges from 13% to 74% depending on the study [11,12]. RA-ILD with UIP has a poor prognosis among connective tissue disease-related ILDs (CTD-ILDs) that is comparable to idiopathic pulmonary fibrosis (IPF) and shares pathophysiologic similarities with IPF [11,13]. For the articular manifestations of RA, there have been many advances in disease-modifying anti-rheumatoid drugs (DMARDs), but the clinical effect of DMARDs on RA-ILD is still controversial [14,15]. While DMARDs may exhibit a treatment response in certain cases of RA-ILD with inflammatory pattern, fibrotic RA-ILD, especially with UIP pattern, tends to be irresponsive to DMARDs [16]. Additionally, there had been no randomized controlled trials on the effectiveness of DMARDs in RA-ILD [17]. Given the poor prognosis, the lack of evidence for treatment, the similarity with IPF, and the emerging concept of progressive pulmonary fibrosis (PPF), interest in using antifibrotic agents, which have shown promising clinical efficacy in IPF patients, for treatment in patients with RA-ILD has been increasing. Two antifibrotic agents, pirfenidone, which inhibits transforming growth factor-β, tumor necrosis factor-α, and platelet-derived growth factor (PDGF) and nintedanib, which inhibits vascular endothelial growth factor, have been known to hinder the pro-fibrotic activity of fibroblasts and fibrocytes [18,19]. Both antifibrotic agents have demonstrated positive clinical effect in patients with IPF by reducing deterioration in lung function and improving life expectancy [20,21,22,23,24].

Studies on the clinical effects and safety of antifibrotic agents in RA-ILD are still needed; therefore, we conducted a meta-analysis of the literature to confirm the clinical effects of pirfenidone and nintedanib on RA-ILD.

## 2. Materials and Methods

This research was conducted according to the Preferred Reporting Items for Systematic Review and Meta-Analyses (PRISMA) statement, and the checklist is described in Table S1. Since this study only used publicly available data, approval from the institutional review board was exempted. The protocol for the systematic review was not registered in a prospective register such as PROSPERO.

### 2.1. Literature Search Strategy

The following databases were searched on 1 June 2023: Cochrane Library databases, EMBASE, KoreaMed, and Ovid MEDLINE (R). Relevant literature published up to the search date, without restrictions on the publication period, was included. There were no language restrictions for the initial search. The extensive literature search focused on the use of two antifibrotic agents, pirfenidone and nintedanib, in patients with RA-ILD. The strategy for the literature search with keywords and combinations is presented in Table S2.

### 2.2. Inclusion Criteria

The Population, Intervention, Comparison, Outcomes and Study (PICOS) design for the inclusion criteria was defined as follows: (1) patients with RA-ILD as the population of interest (P), (2) administration of antifibrotic agents, pirfenidone or nintedanib (I), (3) comparison with the placebo group (C), (4) investigation of the impact of antifibrotic agents on forced vital capacity (FVC) decline as the outcome of interest (O), and (5) randomized controlled trials (S). Additionally, only articles containing complete results and available in full text were included.

### 2.3. Exclusion Criteria

The exclusion criteria were applied as follows: (1) studies on non-human subjects such as animal experiments and laboratory experiments, (2) studies not targeting RA-ILD, (3) studies without comparative interventions between antifibrotic agents and placebo or existing treatments, (4) studies not published in English or Korean, (5) duplicate publications (i.e., publications in other journals with the same content or differences only in publication format), and (6) studies for which the original text could not be obtained.

### 2.4. Primary Outcome

The primary outcome of this study was to evaluate the clinical impact of the antifibrotic agents, nintedanib or pirfenidone, on the disease progression of RA-ILD. The effect of antifibrotic agents was evaluated through the change in FVC over 52 weeks along with the proportion of patients with a decrease in percent predicted FVC of 10% or more over 52 weeks. An expedited deterioration in lung function over time aligns with disease progression and correlates with mortality in various interstitial lung disease [25]. Furthermore, FVC, which is recognized as an established efficacy measure, is also related to the therapeutic effect of antifibrotic agents [26].

### 2.5. Secondary Outcome

Secondary outcomes included (1) changes in the diffusing capacity of the lung for carbon monoxide (DLco) over 52 weeks, (2) all-cause mortality expressed as the number of patients had died over 52 weeks regardless of the cause, and (3) the incidence of overall adverse events, along with severe adverse events and the well-known hurdles associated with the use of antifibrotic agents, namely gastrointestinal side effects.

### 2.6. Data Extraction and Analysis

Two reviewers independently extracted the data from all references. De-duplicated studies were imported into Covidence online software (https://www.covidence.org, accessed on 1 June 2023). Two authors reviewed the titles and abstracts of the de-duplicated studies and chose the relevant studies. Any discrepancies were solved through discussion. Then, two reviewers independently reviewed the full text of the selected article once again. We extracted participant data, inclusion/exclusion criteria, intervention details, and outcome measurements. Any differences in the data extraction were resolved by discussion and/or consultation with a third reviewer.

### 2.7. Assessment of Quality and the Level of Evidence

The quality of selected studies was assessed using the criteria outlined in the Cochrane Handbook for Systematic Reviews of Interventions according to the seven domains: random sequence generation, allocation concealment, blinding of participants and personnel, blinding of outcome assessment, incomplete outcome data, selective reporting, and other biases. Each domain was rated as low, high, or unclear risk. The level of evidence was assessed with the Grading of Recommendations Assessment, Development, and Evaluation (GRADE) approach (GRADEpro, Version 3.6 for Windows, Grade Working group).

### 2.8. Statistical Analysis

The final selected randomized controlled trials (RCTs) were combined using Review Manager 5.4. The heterogeneity of studies was assessed using the Cochrane Q statistic. After evaluating for heterogeneity of studies with the I^2^ statistic, we applied a fixed effects model based on the results. The effect size was measured through the mean difference (MD) for continuous variables, odds ratio or risk ratio for dichotomous variables with a 95% confidence interval (CI). A *p* < 0.05 was considered statistically significant. Due to the small number of included studies, a funnel plot for publication bias was not performed to check for publication bias.

## 3. Results

### 3.1. Literature Review and Selection

**Figure 1:**
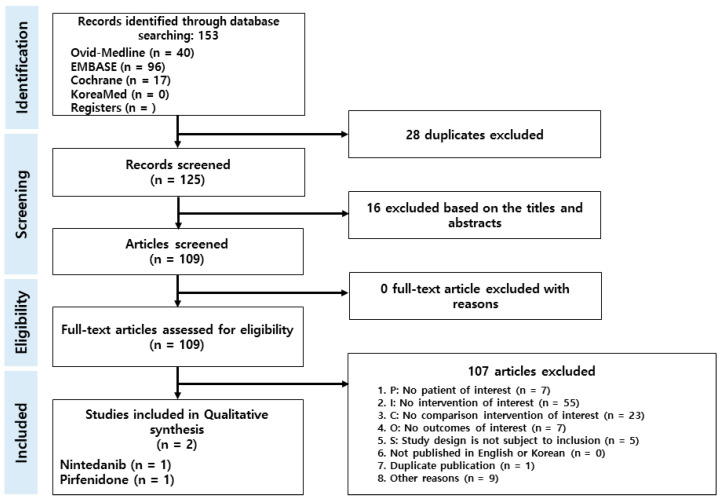
Flow diagram of the Preferred Reporting Items for Systematic Reviews and Meta-Analyses (PRISMA).

A total of 153 articles were identified through database searches, of which 28 were excluded because of duplication. After screening the titles and abstracts, an additional 16 records were removed due to irrelevant publishing types or studies. Thus, a total of 109 articles were used for the study (Figure 1). Out of the 109 full-text articles, 2 qualified for further analysis according to the set inclusion and exclusion criteria.

### 3.2. Characteristics of the Included RCTs

Two eligible RCTs were included in the meta-analysis. The summarized characteristics of the included studies are shown in Table 1. There was a total of two studies administering nintedanib or pirfenidone; however, the composition and dosage of nintedanib or pirfenidone differed in each study.

The study by Matteson et al. [27] was a subgroup analysis for the INBUILD trial and represented the clinical effect of nintedanib in patients with RA-ILD. A total of 89 patients with RA-ILD were included, with 42 of them in the nintedanib group and 47 in the PBO group. Nintedanib was administered at 150 mg twice daily for 52 weeks. In Solomon et al. [28], 63 patients aged 61–74 (mean age, 66 years) were administered oral pirfenidone at 2403 mg per day in divided doses (three 267 mg tablets three times a day). Pirfenidone was titrated to the maximum dose over 14 days (participants took one 267 mg tablet three times a day for days 1–7, two 267 mg tablets a day for days 8–14, and three 267 mg tablets from day 14 onwards) and patients were maintained on the study treatment for 52 weeks.

### 3.3. Effect of Antifibrotic Agents on the Disease Progression in RA-ILD Patients

**Figure 2:**
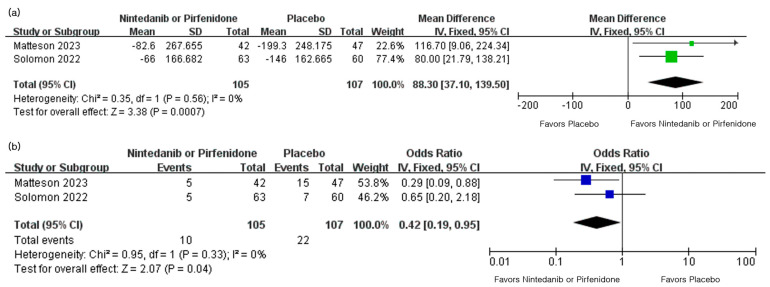
Forest plots for the analysis of the impact of antifibrotic agents on FVC changes in patients with RA-ILD [27,28]: (**a**) forest plot analyzing mean difference of the change in FVC over 52 weeks; (**b**) forest plot comparing the proportion of patients with a decrease of percent predicted FVC by 10% or more over 52 weeks. FVC, forced vital capacity; RA-ILD, rheumatoid arthritis associated interstitial lung disease; SD, standard deviation; IV, inverse variance; CI, confidence interval.

Both RCTs investigated the effect of nintedanib or pirfenidone on the change in FVC in RA-ILD patients. Comparison between the nintedanib or pirfenidone group and the placebo group showed significant differences in FVC. In the study by Matteson et al. [27], the mean (standard error, SE) change in FVC over 52 weeks, which was equivalent to one year, was found to be −82.6 (41.3) mL/year in the nintedanib group and −199.3 (36.2) mL/year in the placebo group. The study conducted by Solomon et al. [28], comparing pirfenidone and placebo, also demonstrated similar results, where the mean (SE) change in FVC over one year was −66 (21) mL/year in the pirfenidone group and −146 (21) mL/year in the placebo group. Since the FVC change values in both studies were presented as means and SE values, the square root of each study’s sample size was multiplied by the SE value to calculate the standard deviation, as a preliminary step for analyzing the effect size. Using the calculated SD values, the analysis of the effect size of antifibrotic agents on FVC change was performed. The result of the analysis revealed that the reduction in FVC was more significant in the placebo group than the antifibrotic agent group (MD, 88.30; 95% CI, 37.10–139.50, *p* = 0.0007). There was no significant heterogeneity between the two included studies (χ^2^ = 0.35, I^2^ = 0%). Both studies also reported the number and proportion of patients with an absolute increase in percent predicted FVC of 10% or more. The meta-analysis results revealed that in the antifibrotic agent group, significantly fewer patients experienced a decrease in percent predicted FVC of 10% or more compared to the placebo group (odds ratio 0.42; 95% CI 0.19–0.95, *p* = 0.04). No significant heterogeneity between the two studies were found (χ^2^ = 0.95, I^2^ = 0%). The results of the meta-analysis and the forest plots are summarized in Figure 2.

### 3.4. Effect of Antifibrotic Agents on Changes in DLco over 52 Weeks

Unfortunately, both RCTs included in the study incorporated the baseline mean DLco in the results, but did not report the changes over the 52-week period. As a result, the analysis of the changes in DLco could not be performed.

### 3.5. All-Cause Mortality over 52 Weeks

**Figure 3:**
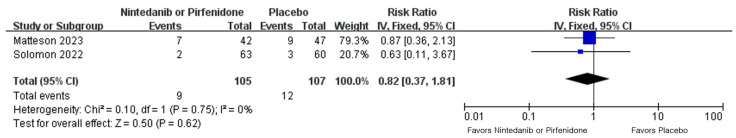
Forest plot for all-cause mortality between the antifibrotic agent group and the placebo group [27,28]. IV, inverse variance; CI, confidence interval.

The results regarding all-cause mortality were reported in both studies. According to the analysis results, there was no significant difference in all-cause mortality between the antifibrotic agent group and the placebo group (risk ratio (RR) 0.82; 95% CI 0.37–1.81, *p* = 0.62) (Figure 3).

### 3.6. Adverse Events over 52-Week Period

**Figure 4:**
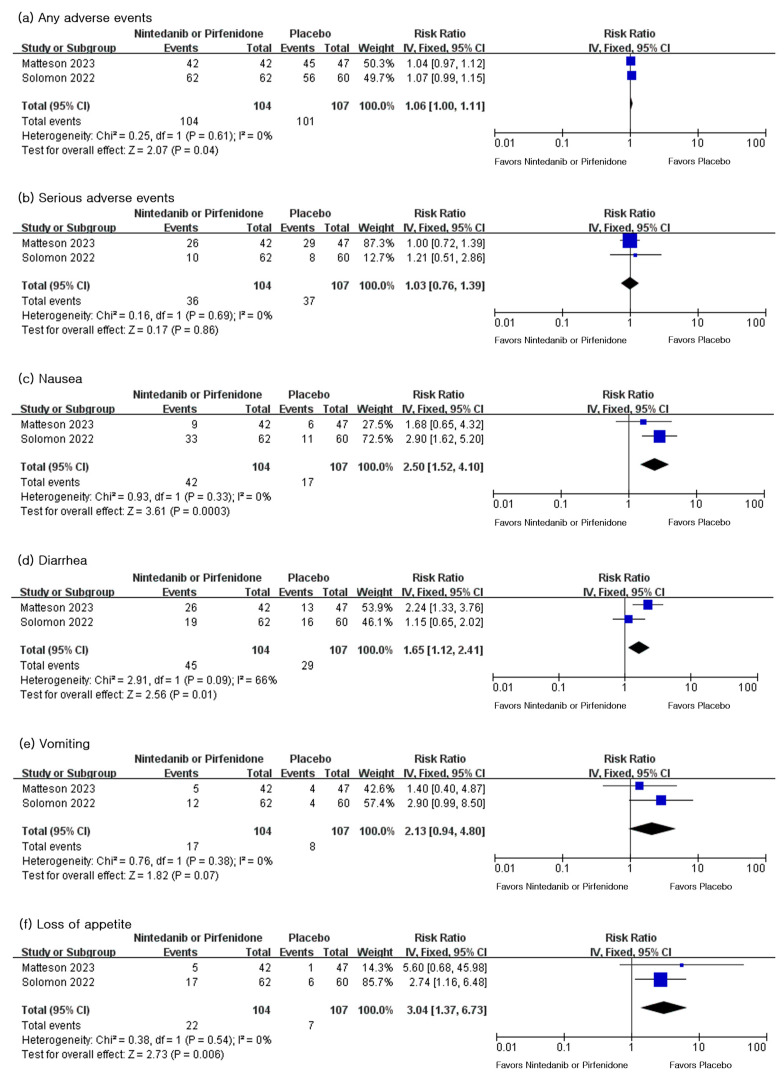
Forest plots for adverse events including overall, serious, and gastrointestinal adverse events [27,28]: forest plots for (**a**) any adverse events, (**b**) serious adverse events, (**c**) nausea, (**d**) diarrhea, (**e**) vomiting, (**f**) loss of appetite. IV, inverse variance; CI, confidence interval.

Adverse events were assessed in both studies. Both studies reported the number of affected patients in each group. The incidence of patients with overall adverse events were higher in the antifibrotic agent group (RR 1.06; 95% CI 1.00–1.11, *p* = 0.04). On the other hand, the incidence of severe adverse events did not show a significant difference between the antifibrotic agent group and the placebo group (RR 1.03; 95% CI 0.76–1.39, *p* = 0.86). The risk of gastrointestinal adverse events differed with statistical significance between the two groups. The risks of nausea (RR 2.5; 95% CI 1.52–4.10, *p* = 0.0003), diarrhea (RR 1.65; 95% CI 1.12–2.41, *p* = 0.01), vomiting (RR 2.13; 95% CI 0.94–4.8, *p* = 0.07), and loss of appetite (RR 3.04; 95% CI 1.37–6.73, *p* = 0.006) were significantly higher in the antifibrotic agent group than the placebo group (Figure 4).

### 3.7. Risk of Bias Assessment

**Figure 5:**
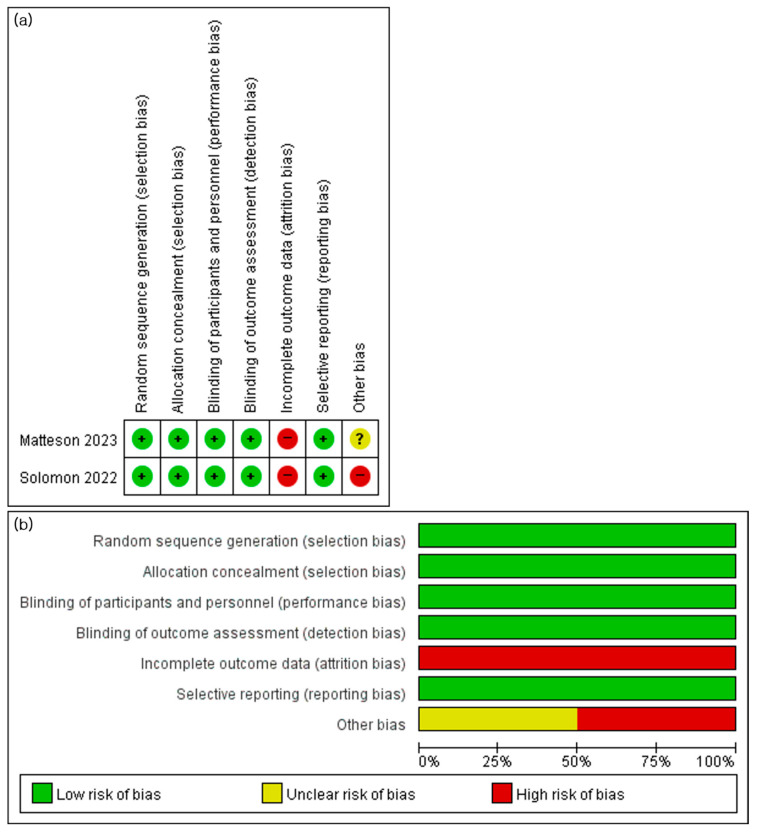
Risk of bias assessment [27,28]: (**a**) risk of bias summary; (**b**) risk of bias graph. Red represents high risk of bias, yellow represents unclear risk of bias, and green represents low risk of bias.

Graphical summary for risk of bias of included studies is presented in Figure 5. Both included RCTs fulfilled random sequence generation, allocation, blinding of participants and personnel, and blinding of outcome assessment. And the risk of selective reporting was deemed low in both studies as they thoroughly reported their outcomes according to the original trial design. However, due to a significant dose reduction or discontinuation rate caused by side effects, a high risk of attrition bias was considered. Additionally, the study by Solomon et al. was deemed to have a potential bias in the results due to early termination resulting from the COVID-19 pandemic and slow recruitment.

### 3.8. Certainty of Evidence for the Effect of Antifibrotic Agents on Disease Progression

**Figure 6:**
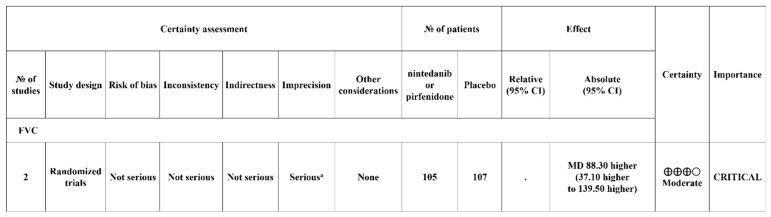
Certainty of evidence for the effect of antifibrotic agents on disease progression by GRADE; ^a^ downgraded for relatively small sample size (N = 105, 107). CI, confidence interval; MD, mean difference.

The certainty of evidence was assessed as per the GRADE approach. Given the relatively small number of patients included in each study, the certainty rating for effect size was considered a “moderate level of evidence”. The assessments and justifications for each GRADE criteria are summarized in Figure 6.

## 4. Discussion

This study included two RCTs involving 212 patients with RA-ILD. Both pirfenidone and nintedanib were shown to reduce the decrease in FVC compared to the placebo group, respectively. Also, in the meta-analysis results, the use of antifibrotic agents was shown to have an effect in reducing FVC decline compared to the placebo group, regardless of the type of antifibrotic agent (MD 88.30; 95% CI, 37.10–139.50). Furthermore, cases where percent predicted FVC decreased by 10% or more in one year, which can be considered as disease progression, occurred less frequently in the antifibrotic agent group (odds ratio 0.42; 95% CI 0.19–0.95, *p* = 0.04). Analysis of the certainty of the effect size was conducted using GRADE, and it was rated as a ‘moderate level of evidence’ considering the relatively small sample size for each RCTs (107 patients in Matteson et al. [27]; 105 patients in Solomon et al. [28]).

Advancements in the treatment of RA have led to a reduction in irreversible joint damage, but there are still many challenges in the treatment of RA-ILD [29]. Although there are conflicting data, concerns remain regarding pulmonary toxicity surrounding the use of immune-modulators (typically represented by DMARDs) to treat RA [30] and a lack of evidence as an effective treatment for ILD [11]. Moreover, UIP is predominant in RA-ILD, unlike other CTD-ILDs, and RA-ILD with UIP has a poorer prognosis than other patterns of ILD such as nonspecific interstitial pneumonia [31,32,33]. Median survival for RA-ILD with UIP pattern has been reported as 3.9 years in the study by Akiyama et al. [29] and 3.5 years in the study by Hakala et al. [34]. While the average survival of IPF varies between 2 and 4 years in different studies and is generally reported around three years [35,36], considering this, RA-ILD with UIP pattern can be considered to exhibit better survival than IPF, but it still carries a poor prognosis. In addition to a poor prognosis, there are more similarities between RA-ILD, especially when accompanied by UIP, and IPF. Several studies have found genetic predisposition as a cause for the high proportion of UIP in RA-ILD, including mutations in genes that are known to be associated with familial interstitial pneumonia, including TERT, RTEL1, and SFTPC [37]. RA-ILD also shares similarities with IPF, including a high prevalence of UIP pattern [38], similar environmental risk factors [39], and a high mortality rate [32]. The MUC5B promotor variant rs35705950 is a strong genetic risk factor for IPF; it is found in more than 50% of patients with IPF and accounts for 30% of the disease-developing risk [40,41,42,43]. Although subsequent study did not show a significant impact of the rs35705950 on transplant-free survival or lung function decline, there has been report indicating that this variant increases the incidence of ILD in RA and the risk of developing UIP pattern in RA-ILD [44,45]. Considering the high proportion of UIP with pathological and physiological similarities to IPF, it seems reasonable to carefully consider antifibrotic agent for the treatment of RA-ILD, especially when UIP is present. However, conducting randomized controlled studies that can serve as evidence for clinical efficacy poses practical challenges and is difficult to carry out. Furthermore, the number of studies conducted for clinical impact of antifibrotic agents in patients with RA-ILD so far is insufficient. Therefore, it is necessary to perform a systematic review with meta-analysis through collecting and integrating relevant studies, increasing the sample size for hypotheses, and enhancing statistical power. Eventually, this study aimed to evaluate the clinical efficacy of antifibrotic agents in patients with RA-ILD through a systematic literature review and meta-analysis.

We selected the change in FVC over 52 weeks and the proportion of patients with a decrease in percent predicted FVC of 10% or more over 52 weeks as the primary endpoints for analyzing whether antifibrotic agents have positive clinical effects in patients with RA-ILD. The degree of disease progression of ILDs has been comprehensively evaluated with the assessment of symptoms, the degree of lung function decline, and the worsening of fibrosis in HRCT [46]. FVC and DLco have been widely used to evaluate the degree of decline in lung function and as indicators to establish treatment strategies in patients with ILD [46,47]. Previous studies have shown that a decline in FVC in ILD patients reflects disease progression and is a significant prognostic factor associated with mortality [48,49,50,51]. Based on these findings, the change in FVC has been gaining prominence as a prognostic indicator in recent guidelines for IPF and progressive fibrosing ILD, and serves as the primary clinical trial endpoint in studies assessing the clinical efficacy of antifibrotic agents in patients with RA-ILD [27,28,52]. Solomon et al. [48] demonstrated in their study that an absolute decline of 10% or more in percent predicted FVC in patients with RA-ILD was associated with increased mortality. Given that an increase in mortality can be considered evidence of the disease progression, an absolute decrease in percent predicted FVC by 10% or more was included in the primary outcome. Diffusing capacity of the lung for carbon monoxide (DLco) is also a helpful measure for evaluating the degree of disease progression and is a screening tool for early or preclinical ILDs [46]. However, a comparative analysis of DLco was not performed in the two RCTs included in this study.

In our study, since antifibrotic agents reduced both the decline in FVC and the proportion of percent predicted FVC decreasing by 10% or more in RA-ILD, clinical efficacy in delaying the disease progression could be positively considered. However, generalizing the effects of antifibrotic agents on CTD-ILD and other fibrosing ILDs beyond RA-ILD is challenging. Given the limited number of included studies and patients, as well as the results being confined to RA-ILD, further research on the effects of antifibrotic agents in fibrosing ILD, including other CTD-ILDs, should be conducted.

From the perspective of mortality, this review did not demonstrate the benefit of antifibrotic agents in patients with RA-ILD. This differs from the results in previous studies on IPF where pirfenidone and nintedanib showed a positive impact on survival [53,54,55]. Since there is a lack of RCTs evaluating the long-term clinical impact of antifibrotic agents in RA-ILD to date, further assessment through additional studies is necessary. On the other hand, gastrointestinal adverse events such as nausea, diarrhea, vomiting, and loss of appetite, showed a higher frequency in the antifibrotic agent group, consistent with previous research findings in IPF [56,57].

Our study has several limitations. First, the number of included studies was small, making it difficult to generalize the results. However, since the total number of patients with RA-ILD included in the analysis was 212, which is not a small number, and the included RCTs were of good quality, the results cannot be seen as exaggerated. Second, this study analyzed the change in FVC, and analysis for other indices such as DLco and mortality were not conducted. Since this study focused on whether antifibrotic agents could reduce the rate of the disease progression, only the effect on FVC was analyzed. Nevertheless, as the change in FVC is a representative outcome for disease progression in patients with ILD, it would not be insufficient to show the clinical effect of antifibrotic agents in RA-ILD patients. Furthermore, as mentioned above, the two studies included in the meta-analysis only examined baseline DLco values without comparing changes over 52 weeks, making it impossible to perform an analysis on this aspect. Third, our study did not implement a funnel plot to evaluate publication bias. Because the number of included studies was less than 10, the asymmetry analysis of the funnel plot was limited. Additional research, including larger-scale studies and additional clinical results such as DLco, parameters from the six-minute walk test, imaging finding beyond FVC, is needed to establish evidence for the therapeutic feasibility of antifibrotic agents in RA-ILD.

## 5. Conclusions

This meta-analysis showed that current antifibrotic agents including nintedanib and pirfenidone may have clinical utility in RA-ILD. In actual clinical practice, the use of antifibrotic agents could be considered for RA-ILD patients. Further research, including a greater number of studies, is needed to confirm the clinical benefit of antifibrotic agents in RA-ILD.

## Figures and Tables

**Table 1:** Summary of characteristics of the included studies and outcomes.

Study	Matteson et al., 2023 [27]		Solomon et al., 2022 [28]	
Published journal	Clinical Rheumatology		The Lancet Respiratory Medicine	
Study design	Double-blind, randomized, placebo-controlled, phase 3 trial (INBUILD)		Double-blind, randomized, placebo-controlled, parallel-group, phase 2 trial (TRAIL1)	
Intervention group	Nintedanib (*n* = 42)	Placebo (*n* = 47)	Pirfenidone (*n* = 63)	Placebo (*n* = 60)
Age, years	66.9 (9.6)		66.0 (61.0–74.0)	69.5 (63.5–74.5)
Male, n (%)	36 (60.7)		38 (60)	39 (65)
Intervention	Nintedanib 150 mg twice a day	Placebo	Pirfenidone three 267 mg tablets three times a day	Placebo
Study duration, weeks	52		52	
Baseline FVC, L	-	-	2.6 (0.8)	2.6 (0.8)
Baseline FVC, % predicted	71.5 (16.2)		69.4 (14.8)	70.4 (14.2)
Baseline DLco, % predicted	47.7 (15.6)		50.0 (12.6)	47.6 (12.8)
Outcome	FVC ^a,b,c^ Acute exacerbation Mortality Hospitalization Progression of ILD ^e^ or death Safety and Tolerability		FVC ^a,b^ Progression by OMERACT ^d^ Mortality Dyspnea-12 questionnaire Hospitalization Safety and Tolerability	

Data are presented as either the mean (SD) or n (%). ^a^. Rate of FVC decline (mL/year); ^b^. The proportion of patients with an absolute decline in FVC ≥ 10% (% predicted); ^c^. The proportion of patients with a relative decline in FVC ≥ 10% (% predicted); ^d^. ≥10% relative decline in FVC or ≥5% to <10% relative decline in FVC and ≥15% relative decline in DLco; ^e^. Defined as an absolute decline from baseline in FVC ≥ 10% (% predicted); FVC, forced vital capacity; DLco, diffusion capacity of the lung for carbon monoxide; HRCT, high-resolution computed tomography; OMERACT, outcome measures for rheumatoid arthritis clinical trials.

## Data Availability

All the data are contained within the manuscript.

## Supplementary Materials

The following supporting information can be downloaded at https://www.mdpi.com/article/10.3390/life13122318/s1. Table S1: PRISMA 2020 Checklist; Table S2: The strategy for the literature search with keywords and combinations for antifibrotic agents.

## References

[1] Smolen J.S., Aletaha D., McInnes I.B. (2016). Rheumatoid arthritis. Lancet.

[2] Cojocaru M., Cojocaru I.M., Silosi I., Vrabie C.D., Tanasescu R. (2010). Extra-articular Manifestations in Rheumatoid Arthritis. Maedica.

[3] Farquhar H., Vassallo R., Edwards A.L., Matteson E.L. (2019). Pulmonary Complications of Rheumatoid Arthritis. Semin. Respir. Crit. Care Med..

[4] Wilsher M., Voight L., Milne D., Teh M., Good N., Kolbe J., Williams M., Pui K., Merriman T., Sidhu K. (2012). Prevalence of airway and parenchymal abnormalities in newly diagnosed rheumatoid arthritis. Respir. Med..

[5] Norton S., Koduri G., Nikiphorou E., Dixey J., Williams P., Young A. (2013). A study of baseline prevalence and cumulative incidence of comorbidity and extra-articular manifestations in RA and their impact on outcome. Rheumatology.

[6] Hyldgaard C., Hilberg O., Pedersen A.B., Ulrichsen S.P., Lokke A., Bendstrup E., Ellingsen T. (2017). A population-based cohort study of rheumatoid arthritis-associated interstitial lung disease: Comorbidity and mortality. Ann. Rheum. Dis..

[7] Doyle T.J., Lee J.S., Dellaripa P.F., Lederer J.A., Matteson E.L., Fischer A., Ascherman D.P., Glassberg M.K., Ryu J.H., Danoff S.K. (2014). A roadmap to promote clinical and translational research in rheumatoid arthritis-associated interstitial lung disease. Chest.

[8] Bongartz T., Nannini C., Medina-Velasquez Y.F., Achenbach S.J., Crowson C.S., Ryu J.H., Vassallo R., Gabriel S.E., Matteson E.L. (2010). Incidence and mortality of interstitial lung disease in rheumatoid arthritis: A population-based study. Arthritis Rheum..

[9] Olson A.L., Swigris J.J., Sprunger D.B., Fischer A., Fernandez-Perez E.R., Solomon J., Murphy J., Cohen M., Raghu G., Brown K.K. (2011). Rheumatoid arthritis-interstitial lung disease-associated mortality. Am. J. Respir. Crit. Care Med..

[10] Raimundo K., Solomon J.J., Olson A.L., Kong A.M., Cole A.L., Fischer A., Swigris J.J. (2019). Rheumatoid Arthritis-Interstitial Lung Disease in the United States: Prevalence, Incidence, and Healthcare Costs and Mortality. J. Rheumatol..

[11] Cassone G., Manfredi A., Vacchi C., Luppi F., Coppi F., Salvarani C., Sebastiani M. (2020). Treatment of Rheumatoid Arthritis-Associated Interstitial Lung Disease: Lights and Shadows. J. Clin. Med..

[12] Suda T. (2015). Up-to-Date Information on Rheumatoid Arthritis-Associated Interstitial Lung Disease. Clin. Med. Insights Circ. Respir. Pulm. Med..

[13] Nurmi H.M., Purokivi M.K., Karkkainen M.S., Kettunen H.P., Selander T.A., Kaarteenaho R.L. (2016). Variable course of disease of rheumatoid arthritis-associated usual interstitial pneumonia compared to other subtypes. BMC Pulm. Med..

[14] Nakashita T., Ando K., Kaneko N., Takahashi K., Motojima S. (2014). Potential risk of TNF inhibitors on the progression of interstitial lung disease in patients with rheumatoid arthritis. BMJ Open.

[15] Picchianti Diamanti A., Markovic M., Argento G., Giovagnoli S., Ricci A., Lagana B., D’Amelio R. (2017). Therapeutic management of patients with rheumatoid arthritis and associated interstitial lung disease: Case report and literature review. Ther. Adv. Respir. Dis..

[16] Song J.W., Lee H.K., Lee C.K., Chae E.J., Jang S.J., Colby T.V., Kim D.S. (2013). Clinical course and outcome of rheumatoid arthritis-related usual interstitial pneumonia. Sarcoidosis Vasc. Diffuse Lung Dis..

[17] Bendstrup E., Moller J., Kronborg-White S., Prior T.S., Hyldgaard C. (2019). Interstitial Lung Disease in Rheumatoid Arthritis Remains a Challenge for Clinicians. J. Clin. Med..

[18] Maher T.M., Strek M.E. (2019). Antifibrotic therapy for idiopathic pulmonary fibrosis: Time to treat. Respir. Res..

[19] Yang M., Wu Y., Liu X., Zhao C., Li T., Li T., Zhang X., Jiang H., Mao B., Liu W. (2023). Efficacy and safety of antifibrotic agents in the treatment of CTD-ILD and RA-ILD: A systematic review and meta-analysis. Respir. Med..

[20] van Manen M.J.G., Birring S.S., Vancheri C., Vindigni V., Renzoni E., Russell A.M., Wapenaar M., Cottin V., Wijsenbeek M.S. (2017). Effect of pirfenidone on cough in patients with idiopathic pulmonary fibrosis. Eur. Respir. J..

[21] Fisher M., Nathan S.D., Hill C., Marshall J., Dejonckheere F., Thuresson P.O., Maher T.M. (2017). Predicting Life Expectancy for Pirfenidone in Idiopathic Pulmonary Fibrosis. J. Manag. Care Spec. Pharm..

[22] Richeldi L., Cottin V., du Bois R.M., Selman M., Kimura T., Bailes Z., Schlenker-Herceg R., Stowasser S., Brown K.K. (2016). Nintedanib in patients with idiopathic pulmonary fibrosis: Combined evidence from the TOMORROW and INPULSIS((R)) trials. Respir. Med..

[23] Ley B., Swigris J., Day B.M., Stauffer J.L., Raimundo K., Chou W., Collard H.R. (2017). Pirfenidone Reduces Respiratory-related Hospitalizations in Idiopathic Pulmonary Fibrosis. Am. J. Respir. Crit. Care Med..

[24] Collard H.R., Richeldi L., Kim D.S., Taniguchi H., Tschoepe I., Luisetti M., Roman J., Tino G., Schlenker-Herceg R., Hallmann C. (2017). Acute exacerbations in the INPULSIS trials of nintedanib in idiopathic pulmonary fibrosis. Eur. Respir. J..

[25] Schmidt S.L., Tayob N., Han M.K., Zappala C., Kervitsky D., Murray S., Wells A.U., Brown K.K., Martinez F.J., Flaherty K.R. (2014). Predicting pulmonary fibrosis disease course from past trends in pulmonary function. Chest.

[26] du Bois R.M., Nathan S.D., Richeldi L., Schwarz M.I., Noble P.W. (2012). Idiopathic pulmonary fibrosis: Lung function is a clinically meaningful endpoint for phase III trials. Am. J. Respir. Crit. Care Med..

[27] Matteson E.L., Aringer M., Burmester G.R., Mueller H., Moros L., Kolb M. (2023). Effect of nintedanib in patients with progressive pulmonary fibrosis associated with rheumatoid arthritis: Data from the INBUILD trial. Clin. Rheumatol..

[28] Solomon J.J., Danoff S.K., Woodhead F.A., Hurwitz S., Maurer R., Glaspole I., Dellaripa P.F., Gooptu B., Vassallo R., Cox P.G. (2023). Safety, tolerability, and efficacy of pirfenidone in patients with rheumatoid arthritis-associated interstitial lung disease: A randomised, double-blind, placebo-controlled, phase 2 study. Lancet Respir. Med..

[29] Akiyama M., Kaneko Y. (2022). Pathogenesis, clinical features, and treatment strategy for rheumatoid arthritis-associated interstitial lung disease. Autoimmun. Rev..

[30] Roubille C., Haraoui B. (2014). Interstitial lung diseases induced or exacerbated by DMARDS and biologic agents in rheumatoid arthritis: A systematic literature review. Semin. Arthritis Rheum..

[31] Lee H.K., Kim D.S., Yoo B., Seo J.B., Rho J.Y., Colby T.V., Kitaichi M. (2005). Histopathologic pattern and clinical features of rheumatoid arthritis-associated interstitial lung disease. Chest.

[32] Kim E.J., Elicker B.M., Maldonado F., Webb W.R., Ryu J.H., Van Uden J.H., Lee J.S., King T.E., Collard H.R. (2010). Usual interstitial pneumonia in rheumatoid arthritis-associated interstitial lung disease. Eur. Respir. J..

[33] Tsuchiya Y., Takayanagi N., Sugiura H., Miyahara Y., Tokunaga D., Kawabata Y., Sugita Y. (2011). Lung diseases directly associated with rheumatoid arthritis and their relationship to outcome. Eur. Respir. J..

[34] Hakala M. (1988). Poor prognosis in patients with rheumatoid arthritis hospitalized for interstitial lung fibrosis. Chest.

[35] American Thoracic S., European Respiratory S. (2002). American Thoracic Society/European Respiratory Society International Multidisciplinary Consensus Classification of the Idiopathic Interstitial Pneumonias. This joint statement of the American Thoracic Society (ATS), and the European Respiratory Society (ERS) was adopted by the ATS board of directors, June 2001 and by the ERS Executive Committee, June 2001. Am. J. Respir. Crit. Care Med..

[36] Kim D.S., Collard H.R., King T.E. (2006). Classification and natural history of the idiopathic interstitial pneumonias. Proc. Am. Thorac. Soc..

[37] Juge P.A., Borie R., Kannengiesser C., Gazal S., Revy P., Wemeau-Stervinou L., Debray M.P., Ottaviani S., Marchand-Adam S., Nathan N. (2017). Shared genetic predisposition in rheumatoid arthritis-interstitial lung disease and familial pulmonary fibrosis. Eur. Respir. J..

[38] Kim E.J., Collard H.R., King T.E. (2009). Rheumatoid arthritis-associated interstitial lung disease: The relevance of histopathologic and radiographic pattern. Chest.

[39] Kelly C.A., Saravanan V., Nisar M., Arthanari S., Woodhead F.A., Price-Forbes A.N., Dawson J., Sathi N., Ahmad Y., Koduri G. (2014). Rheumatoid arthritis-related interstitial lung disease: Associations, prognostic factors and physiological and radiological characteristics--a large multicentre UK study. Rheumatology.

[40] Seibold M.A., Wise A.L., Speer M.C., Steele M.P., Brown K.K., Loyd J.E., Fingerlin T.E., Zhang W., Gudmundsson G., Groshong S.D. (2011). A common MUC5B promoter polymorphism and pulmonary fibrosis. N. Engl. J. Med..

[41] Fingerlin T.E., Murphy E., Zhang W., Peljto A.L., Brown K.K., Steele M.P., Loyd J.E., Cosgrove G.P., Lynch D., Groshong S. (2013). Genome-wide association study identifies multiple susceptibility loci for pulmonary fibrosis. Nat. Genet..

[42] Noth I., Zhang Y., Ma S.F., Flores C., Barber M., Huang Y., Broderick S.M., Wade M.S., Hysi P., Scuirba J. (2013). Genetic variants associated with idiopathic pulmonary fibrosis susceptibility and mortality: A genome-wide association study. Lancet Respir. Med..

[43] Zhang Y., Noth I., Garcia J.G., Kaminski N. (2011). A variant in the promoter of MUC5B and idiopathic pulmonary fibrosis. N. Engl. J. Med..

[44] Juge P.A., Lee J.S., Ebstein E., Furukawa H., Dobrinskikh E., Gazal S., Kannengiesser C., Ottaviani S., Oka S., Tohma S. (2018). MUC5B Promoter Variant and Rheumatoid Arthritis with Interstitial Lung Disease. N. Engl. J. Med..

[45] Juge P.A., Solomon J.J., van Moorsel C.H.M., Garofoli R., Lee J.S., Louis-Sydney F., Rojas-Serrano J., Gonzalez-Perez M.I., Mejia M., Buendia-Roldan I. (2021). MUC5B promoter variant rs35705950 and rheumatoid arthritis associated interstitial lung disease survival and progression. Semin. Arthritis Rheum..

[46] Kumar D.P. (2021). Assessment and Follow-Up of Interstitial Lung Disease. Indian J. Rheumatol..

[47] Showalter K., Hoffmann A., Rouleau G., Aaby D., Lee J., Richardson C., Dematte J., Agrawal R., Chang R.W., Hinchcliff M. (2018). Performance of Forced Vital Capacity and Lung Diffusion Cutpoints for Associated Radiographic Interstitial Lung Disease in Systemic Sclerosis. J. Rheumatol..

[48] Solomon J.J., Chung J.H., Cosgrove G.P., Demoruelle M.K., Fernandez-Perez E.R., Fischer A., Frankel S.K., Hobbs S.B., Huie T.J., Ketzer J. (2016). Predictors of mortality in rheumatoid arthritis-associated interstitial lung disease. Eur. Respir. J..

[49] Qiu M., Jiang J., Nian X., Wang Y., Yu P., Song J., Zou S. (2021). Factors associated with mortality in rheumatoid arthritis-associated interstitial lung disease: A systematic review and meta-analysis. Respir. Res..

[50] Brown K.K., Martinez F.J., Walsh S.L.F., Thannickal V.J., Prasse A., Schlenker-Herceg R., Goeldner R.G., Clerisme-Beaty E., Tetzlaff K., Cottin V. (2020). The natural history of progressive fibrosing interstitial lung diseases. Eur. Respir. J..

[51] Nasser M., Larrieu S., Si-Mohamed S., Ahmad K., Boussel L., Brevet M., Chalabreysse L., Fabre C., Marque S., Revel D. (2021). Progressive fibrosing interstitial lung disease: A clinical cohort (the PROGRESS study). Eur. Respir. J..

[52] Raghu G., Remy-Jardin M., Richeldi L., Thomson C.C., Inoue Y., Johkoh T., Kreuter M., Lynch D.A., Maher T.M., Martinez F.J. (2022). Idiopathic Pulmonary Fibrosis (an Update) and Progressive Pulmonary Fibrosis in Adults: An Official ATS/ERS/JRS/ALAT Clinical Practice Guideline. Am. J. Respir. Crit. Care Med..

[53] Margaritopoulos G.A., Trachalaki A., Wells A.U., Vasarmidi E., Bibaki E., Papastratigakis G., Detorakis S., Tzanakis N., Antoniou K.M. (2018). Pirfenidone improves survival in IPF: Results from a real-life study. BMC Pulm. Med..

[54] Richeldi L., du Bois R.M., Raghu G., Azuma A., Brown K.K., Costabel U., Cottin V., Flaherty K.R., Hansell D.M., Inoue Y. (2014). Efficacy and safety of nintedanib in idiopathic pulmonary fibrosis. N. Engl. J. Med..

[55] King T.E., Bradford W.Z., Castro-Bernardini S., Fagan E.A., Glaspole I., Glassberg M.K., Gorina E., Hopkins P.M., Kardatzke D., Lancaster L. (2014). A phase 3 trial of pirfenidone in patients with idiopathic pulmonary fibrosis. N. Engl. J. Med..

[56] Jiang C., Huang H., Liu J., Wang Y., Lu Z., Xu Z. (2012). Adverse events of pirfenidone for the treatment of pulmonary fibrosis: A meta-analysis of randomized controlled trials. PLoS ONE.

[57] Chen C.H., Lin H.C., Wang Y.H., Wang C.Y., Lin Y.S., Lai C.C. (2021). The safety of nintedanib for the treatment of interstitial lung disease: A systematic review and meta-analysis of randomized controlled trials. PLoS ONE.

